# The core effector Cce1 is required for early infection of maize by *Ustilago maydis*

**DOI:** 10.1111/mpp.12698

**Published:** 2018-08-16

**Authors:** Denise Seitner, Simon Uhse, Michelle Gallei, Armin Djamei

**Affiliations:** ^1^ Gregor Mendel Institute (GMI), Austrian Academy of Sciences (OEAW), Vienna BioCenter (VBC) Vienna 1030 Austria; ^2^ Institute of Science and Technology Austria Klosterneuburg 3400 Austria

**Keywords:** biotrophic interaction, effector, filamentous fungus, plant pathogen, *Ustilago maydis*, virulence factor, Zea mays

## Abstract

The biotrophic pathogen *Ustilago maydis*, the causative agent of corn smut disease, infects one of the most important crops worldwide – *Zea mays. *To successfully colonize its host, *U. maydis *secretes proteins, known as effectors, that suppress plant defense responses and facilitate the establishment of biotrophy. In this work, we describe the *U. maydis *effector protein Cce1. Cce1 is essential for virulence and is upregulated during infection. Through microscopic analysis and *in vitro* assays, we show that Cce1 is secreted from hyphae during filamentous growth of the fungus. Strikingly, Δ*cce1* mutants are blocked at early stages of infection and induce callose deposition as a plant defense response. Cce1 is highly conserved among smut fungi and the *Ustilago bromivora *ortholog complemented the virulence defect of the SG200Δcce1 deletion strain. These data indicate that Cce1 is a core effector with apoplastic localization that is essential for *U. maydis* to infect its host.

## Introduction

The primary goal of plant pathogenic fungi is to proliferate using the host as a source of nutrients, shelter, and a structure from which to distribute spores. Biotrophic fungi depend on a living host, which has led to the evolution of sophisticated strategies for suppressing host defense and redirecting its metabolic flux. The gall inducing, biotrophic fungus *Ustilago maydis* grows with intra‐ and extracellular hyphae after appressoria‐mediated penetration of the maize epidermis. The intracellular hyphae are encased by the host membrane, forming the biotrophic interface where the exchange of nutrients as well as defense and defense suppression molecules occurs (Brefort et al., 2009; Jones and Dangl, 2006).

To establish biotrophy, *U. maydis* needs to overcome the multilayered defense system of its host, *Z. mays*. PAMP (Pathogen associated molecular pattern)‐triggered immunity (PTI) responses are activated if the pathogen is unable to avoid recognition. The maize response includes apoplastic H_2_O_2_ production, directed secretion of papilla material like callose to block fungal penetration, and induction of defense genes (Cook et al., 2015; Oliveira‐Garcia and Deising, 2016).

Defense suppression is achieved by deploying a subset of secreted molecules, termed effectors, from the fungal hyphae (Sperschneider et al., 2016). Effector proteins secreted through the conventional pathway via the endoplasmatic reticulum and the Golgi apparatus are either localized to the biotrophic interphase (apoplastic effectors) or can be translocated into the plant cell (symplastic effectors) (Lo Presti et al., 2015). Apoplastic effectors have been shown to inhibit extracellular host enzymes, chelate PAMPs to reduce the possibility of host recognition, and degrade host cell walls (de Jonge et al., 2011; Win et al., 2012).

Criteria frequently used for effector prediction are the presence of a secretion signal, upregulation of expression upon host colonization, and a maximum protein size of 300 amino acids (Lo Presti et al., 2015). Furthermore, most effectors lack conserved functional domains as well as orthologs outside the genus (Win et al., 2012). Many effector proteins are cysteine‐rich and presumably able to form disulfide bonds to stabilize their tertiary structure in the harsh oxidative conditions of the plant apoplast (Lanver et al., 2017; Win et al., 2012).

In *U. maydis*, there are 467 putative proteins that are predicted to be secreted. Of these, 43% lack recognizable domains and are expected to play an important role in host colonization (Schuster et al., 2017)*.* These potential effectors are often encoded in one of twelve effector gene clusters (Kämper et al., 2006). Interestingly, individual cluster mutants are affected at distinct developmental steps of biotrophy, therefore the effectors are likely to have separate cellular targets (Brefort et al., 2009; Kämper et al., 2006). In many cases, however, deletion of a single effector or even a whole cluster does not affect *U. maydis *virulence, presumably due to functional redundancy. *U. maydis *also contains core effectors, which are highly conserved among smut fungi and are likely to play a crucial role in the infection process (Lanver et al., 2017).

Only a few individual effectors of *U. maydis* have been functionally characterized. The apoplastic effector Pit2 (Protein involved in tumours 2) inhibits a set of apoplastic maize cysteine proteases, thereby suppressing plant defense responses (Mueller et al., 2013). The core effector Pep1 (Protein essential for penetration 1) also acts in the apoplast and targets a maize‐derived peroxidase, inhibiting the accumulation of H_2_O_2_ released from the plant as a defense response at the site of penetration (Doehlemann et al., 2009). An example of a translocated effector is the chorismate mutase Cmu1*. *This cytoplasmic effector reduces the levels of chorismate, a precursor of the plant defense hormone salicylic acid (Djamei et al., 2011). Tin2 (Tumour inducing 2) also translocates into the plant cell where it enhances the production of anthocyanin to redirect precursors away from lignin biosynthesis (Tanaka et al., 2014). See1 (Seedling efficient effector 1) was the first organ‐specific effector characterized and it reactivates plant DNA synthesis during leaf gall progression (Redkar et al., 2015). The virulence factor ApB73 is crucial for the establishment of biotrophy but its mode of action is not yet understood (Stirnberg and Djamei, 2016).

Here, we characterize Cce1 (**C**ysteine‐rich **c**ore **e**ffector 1), a cysteine‐rich core effector of *U. maydis*. Cce1 is upregulated during the biotrophic stage, secreted from the fungus, highly conserved on the sequence level in other smut fungi, and indispensable at the onset of infection.

## Results

### Identification of Cce1

**Figure 1:**
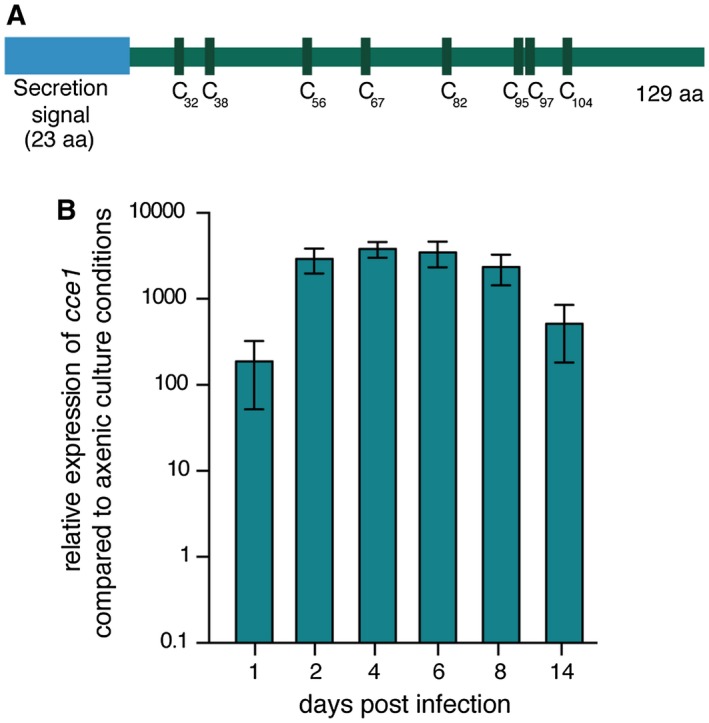
Cce1 protein structure and its transcriptional regulation during biotrophy. (A) Schematic representation of Cce1. The 129 amino acid protein consists of a putative N‐terminal secretion signal (amino acid 1‐23) and eight cysteine residues in the center (C32, 38, 56, 67, 82, 95, 97, 104). (B) Transcript levels of *cce1* measured at different time points during biotrophic development of FB1/FB2 strains infecting *Z. mays*. Expression levels were normalized to the constitutive gene *peptidyl‐prolyl isomerase* (*ppi*). Three biological replicates were analyzed, error bars indicate standard deviation.

Although many putative effectors have been previously found in clusters in the *U. maydis* genome, we focused on highly conserved genes encoding putative effectors outside of these clusters. Here we identified the *cce1* gene (UMAG_12197) located on chromosome 7 of *U. maydis*. The gene located upstream of *cce1* is predicted to be related to the B56‐delta regulatory subunit of protein phosphatase 2A, and the downstream neighboring gene is related to SGS1 (Slow Growth Suppressor 1), encoding a DNA helicase. The c*ce1* gene itself contains one intron (position 240 to 326) and encodes a protein of 129 amino acids. The Cce1 protein was predicted to be secreted by SignalP (Petersen et al., 2011), with the putative N‐terminal secretion signal expected to be cleaved after 23 amino acids. Besides the secretion signal, no known protein domains are found within Cce1. The mature protein contains eight cysteines, resulting in four possible disulfide bonds that could stabilize the protein in the harsh conditions of the apoplast after secretion (Fig. 1A). Prediction of apoplastic localization was tested using ApoplastP (Sperschneider et al., 2017), which showed that Cce1 has a 57% probability of being localized to the apoplast (Table 1).

**Table 1:** Secretion prediction, apoplastic localisation prediction and identity to Cce1 of Cce1 orthologs.

Organism	Gene ID	Secretion prediction	Predicted secretion signal cleavage site	Apoplastic localisation prediction	Identity to Cce1	Similarity to Cce1
*U. maydis*	UMAG_12197	Yes	VRA‐AT	Yes (57%)		
*U. hordei*	UHOR_04531	Yes	VEA‐KT	Yes (64%)	59%	67%
*U. bromivora*	UBRO_04531	Yes	VEA‐GT	Yes (65%)	59%	66%
*S. scitamineum*	SPSC_06609	Yes	VEA‐AT	No (51%)	65%	74%
*S. reilianum*	sr13927	Yes	VEA‐KT	Yes (84%)	61%	71%
*M. antarcticus*	PAN0_010d4106	Yes	VVA‐ST	Yes (74%)	60%	74%

Effector genes are strongly upregulated in transcripts from biotrophic stages compared to transcripts from axenically grown cultures (Skibbe et al., 2010). We performed quantitative real‐time PCR (qRT‐PCR) analysis and found that *cce1* is transcriptionally upregulated more than 3000‐fold during biotrophy, indicating that Cce1 is required during the biotrophic growth phase of the fungus (Fig. 1B).

### Cce1 is required for virulence of *U. maydis*

**Figure 2:**
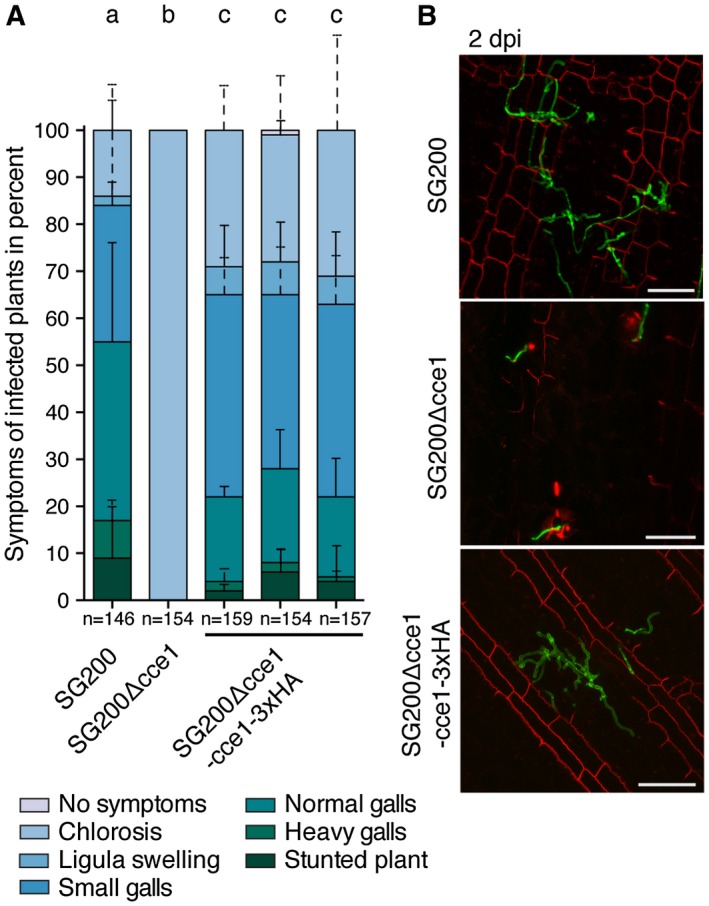
Symptom rating and *in planta* growth of *U. maydis *hyphae. (A) Symptom rating of the *U. maydis *strain SG200Δcce1 in comparison to the progenitor strain SG200 and three individual SG200Δcce1‐cce1‐3xHA complementation strains 12 days post infection (dpi). Mean and standard deviation of relative counts from four replicates are displayed. For clarity, only positive error bars are shown. n = number of plants scored. Significant differences between the strains are indicated by a, b, c. p‐values calculated by Fisher’s exact test, MTC by Benjamini‐Hochberg algorithm, α = 0.05. (B) Seven‐day‐old *Z. mays *seedlings were infected with *U. maydis *strains, leaves were harvested two days post infection (dpi). Fungal hyphae were visualized by WGA‐AF488 (green), plant cell walls stained with propidium iodide, and observed via confocal microscopy. Maximum projections of Z‐stacks are shown; scale bar = 50 μm.

To determine whether deletion of *cce1 *had an impact on virulence of *U. maydis, *seven‐day old *Z. mays *seedlings (cultivar EGB) were infected with either an SG200 *cce1 *deletion strain (SG200Δcce1), the solopathogenic progenitor strain SG200, or the ectopic complementation strain SG200Δcce1‐cce1‐3xHA. Scoring of disease symptoms showed that the *cce1 *deletion strain is no longer able to induce symptoms beyond weak chlorosis, possibly due to local hypersensitive responses restricting the pathogen. Ectopic complementation partially restored the virulence phenotype, including gall induction (Fig. 2A).

To investigate if Cce1 is needed for growth of *U. maydis*, the mutant strains were tested for their ability to form filaments, a pre‐requisite for infection, as well as growth under different conditions including nutrient deprivation, and oxidative, salt, and cell wall stress (Supporting Information Fig.S1). We did not observe any growth differences between SG200, SG200Δcce1, and SG200Δcce1‐cce1‐3xHA, indicating that Cce1 is specifically needed during the biotrophic phase of *U. maydis*.

To elucidate at which stage of infection the SG200Δcce1 strain is impaired, we followed its growth *in planta*. For this, *Z. mays *seedlings were infected with either SG200*, *SG200Δcce1, or the complementation strain SG200Δcce1‐cce1‐3xHA. In comparison to SG200 and the complementation strain, the development of SG200Δcce1 was arrested at the onset of infection (Fig. 2B and Supporting Information Fig. S2). In addition to its use as a cell wall stain, propidium iodide can stain nucleic acids if the integrity of the plasma membrane is disrupted (Lo Presti et al., 2017). Consistent with the macroscopic chlorotic symptoms we observed, cells next to SG200Δcce1 often showed staining of internal structures indicating local hypersensitive responses leading to cellular disintegration and propidium iodide staining of nucleic acids (Fig. 2B and Supporting Information Fig. S2). This again suggests that Cce1 is essential for virulence of *U. maydis* during its infection of maize.

To exclude the possibility that Cce1 is involved in the formation of penetration structures, we quantified the formation of appressoria and penetration structures with a microscopy‐based assay (Mendoza‐Mendoza et al., 2009). We compared appressorial marker induction and penetration efficiency of a fluorescently labelled *cce1* mutant strain to the progenitor strain. No significant differences between the two strains were observed, demonstrating that appressoria formation is independent of Cce1 and the SG200Δcce1‐AM1 strain is still able to form penetration structures (Supporting Information Fig. S3).

Taken together, these results show that Cce1 is not involved in stress responses or appressoria formation of *U. maydis*, but is essential for the virulence of the fungus early after the initial penetration event.

### Cce1 is an effector

**Figure 3:**
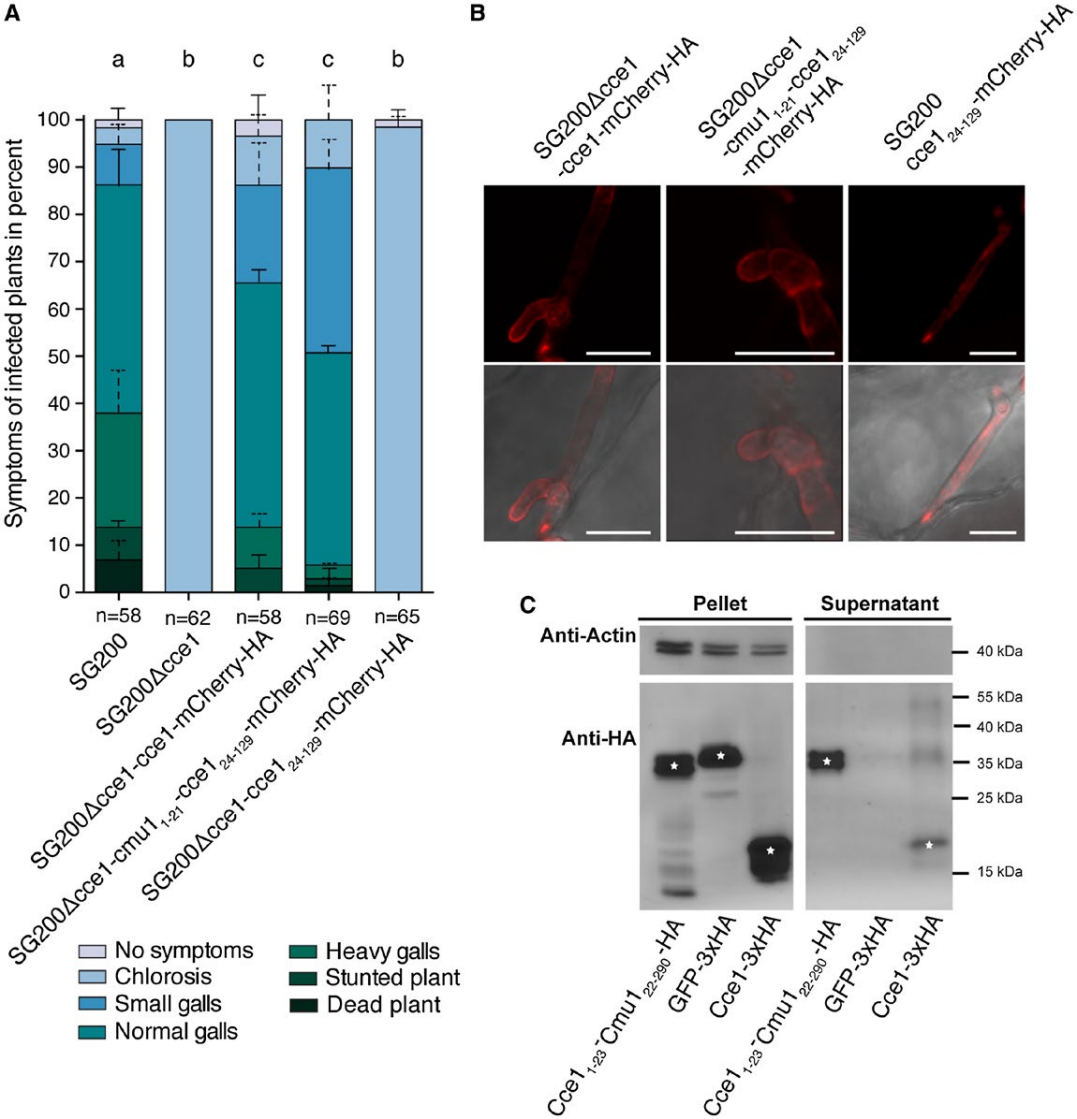
Disease symptom rating and secretion of Cce1. (A) Seven‐day‐old *Z. mays *seedlings were infected with the *U. maydis *strains SG200, SG200Δcce1, and the three complementation strains SG200Δcce1‐cce1‐mCherry‐HA, SG200Δcce1‐cmu1_1‐21_‐cce1_24‐129_‐mCherry‐HA, and SG200Δcce1‐cce1_24‐129_‐mCherry‐HA. Disease symptoms were scored twelve days post infection (dpi). Mean and standard deviation of relative counts from two replicates are displayed. For clarity, only positive error bars are shown. n = number of plants scored. Significant differences between the strains are indicated by a, b, c. p‐values calculated by Fisher’s exact test, MTC by Benjamini‐Hochberg algorithm. α = 0.05 (B) Localization of Cce1‐mCherry‐HA fusion proteins with and without secretion signal. The Cce1‐mCherry‐HA fusion proteins containing endogenous Cce1 signal peptide (left) as well as the same construct containing the signal peptide from Cmu1 (middle) are localized mainly at the periphery of the fungus. The Cce1‐mCherry‐HA version without secretion signal (right) is located in the cytoplasm of the fungus. Photomicrographs were taken seven days post infection. Upper row: mCherry fluorescence (red); lower row: overlay of mCherry channel and brightfield. Scale bar = 10 μm. (C) Secretion of Cce1 in axenic culture. Cce1‐3xHA protein as well as Cce1_1‐23_‐Cmu1_22‐290_‐HA was detected by immunoblotting in the pellet as well as in the supernatant of the strain AB33 P_otef_‐cce1‐3xHA and AB33 P_otef_‐cce1_1‐23_‐cmu1_22‐290_‐HA, respectively, whereas cytosolic GFP‐3xHA as well as actin was only detected in the cell pellet fraction. White asterisks on the immunoblot bands indicate the expected band size for the respective immunolabelled proteins.

As one hallmark of effector proteins is that they are secreted, we tested if Cce1 is secreted, and whether its secretion is essential for virulence. We generated *cce1 *constructs with C‐terminal *mCherry‐HA *tags under control of the native promoter that either lack the secretion signal (*cce1_24_*_‐_ *_129_*) or were substituted by the signal peptide of Cmu1 (*cmu1_1‐21_‐cce1_24‐129_*), a well characterized, secreted effector of *U. maydis (*Djamei et al., 2011)*, *and ectopically integrated them into SG200Δcce1. The strain expressing *cce1 *fused to *mCherry‐HA *under control of the *cce1 *promoter was used to test if Cce1‐mCherry‐HA fusion proteins are still functional. Assessment of pathogenicity revealed that strains containing a secretion signal, either from Cce1 itself (SG200Δcce1‐cce1‐mCherry‐HA) or from Cmu1 (SG200Δcce1‐cmu1_1‐21_‐cce1_24‐129_‐mCherry‐HA), can partially complement the virulence defect of the SG200Δcce1 strain. The strain lacking the signal peptide (SG200Δcce1‐cce1_24‐129_‐mCherry‐HA) was not able to infect the plant and did not cause symptoms beyond chlorosis, indicating that secretion of Cce1 is essential for its function (Fig. 3A).

To determine the localization of the Cce1‐mCherry‐HA fusion protein with or without secretion signals, we performed confocal laser scanning microscopy on infected maize leaf sections. As SG200Δcce1 cannot be complemented with Cce1_24‐129_‐mCherry‐HA, this construct was transformed into SG200 (SG200‐cce1_24‐129_‐mCherry‐HA) to compare its localization pattern with the other mCherry fusion proteins. The fusion proteins containing a secretion signal were tested using the same SG200Δcce1 complementation strains as in the previous virulence assay. The fusion proteins Cce1‐mCherry‐HA as well as Cmu1_1‐21_‐Cce1_24‐129_‐mCherry‐HA localized mainly at the surroundings of the fungal hyphae in the biotrophic interface, indicating that these proteins are secreted. In contrast, in the SG200 strain expressing Cce1_24‐129_, the mCherry signal was detected in the cytoplasm of the fungus and not at the surroundings (Fig. 3B and Supporting Information Fig. S4). In addition, plasmolysis was induced to increase the area of the biotrophic interphase. Consistent with the previous findings, the fluorescent signal of Cce1‐mCherry‐HA is present in the surroundings of the fungal hyphae (Supporting Information Fig. S5). Immunoblotting of the Cce1‐mCherry‐HA fusion proteins revealed that these proteins are either cleaved by the fungus or the plant or degraded during the experimental procedure. Therefore, we cannot exclude that cleavage products are taken up by the plant cell (Supporting Information Fig. S6). In conclusion, these results suggest that the predicted Cce1 signal peptide enables the secretion of Cce1 to the biotrophic interface.

To further support our phenotyping and microscopy data, secretion of Cce1‐3xHA was tested in axenic culture of the *U. maydis *strain AB33. The transgenic AB33 strain carries a heterodimeric transcription factor under control of a nitrogen‐inducible promoter to trigger filament formation, thereby mimicking a developmental stage usually induced only on the plant leaf upon infection (Brachmann et al., 2001). AB33 was transformed with *p123‐otef:cce1*‐*3xHA *under control of the constitutively active *otef*‐promoter. To confirm the functionality of Cce1’s predicted secretion signal independent of the Cce1 protein, it was fused to Cmu1_22‐290_ (Cce1_1‐23_‐Cmu1_22‐290_‐HA), while GFP‐3xHA was used as a negative control. To exclude that detection of Cce1‐3xHA was due to cellular lysis and not from secretion, all samples, including supernatants, were also tested for the presence or absence of actin as a cytoplasmic marker. Immunoblotting of the proteins revealed that GFP‐3xHA, as well as actin, could only be detected in the cell pellet, whereas Cce1‐3xHA and Cce1_1‐23_‐Cmu1_22‐290_‐HA were found in the supernatant. This finding indicates that the secretion signal of Cce1 is functional and Cce1‐3xHA is secreted from the fungus in axenic culture (Fig. 3C).

We have shown that Cce1 is secreted from the fungus into supernatants when constitutively expressed, that Cce1 without a secretion signal is not able to complement the virulence defect of the deletion strain, and that Cce1‐mCherry‐HA fusion proteins are secreted from the fungal hyphae. Taken together, this indicates that Cce1 is an essential effector for *Ustilago maydis *and that secretion is inevitable for its function.

### SG200Δcce1 induces early plant defense response

**Figure 4:**
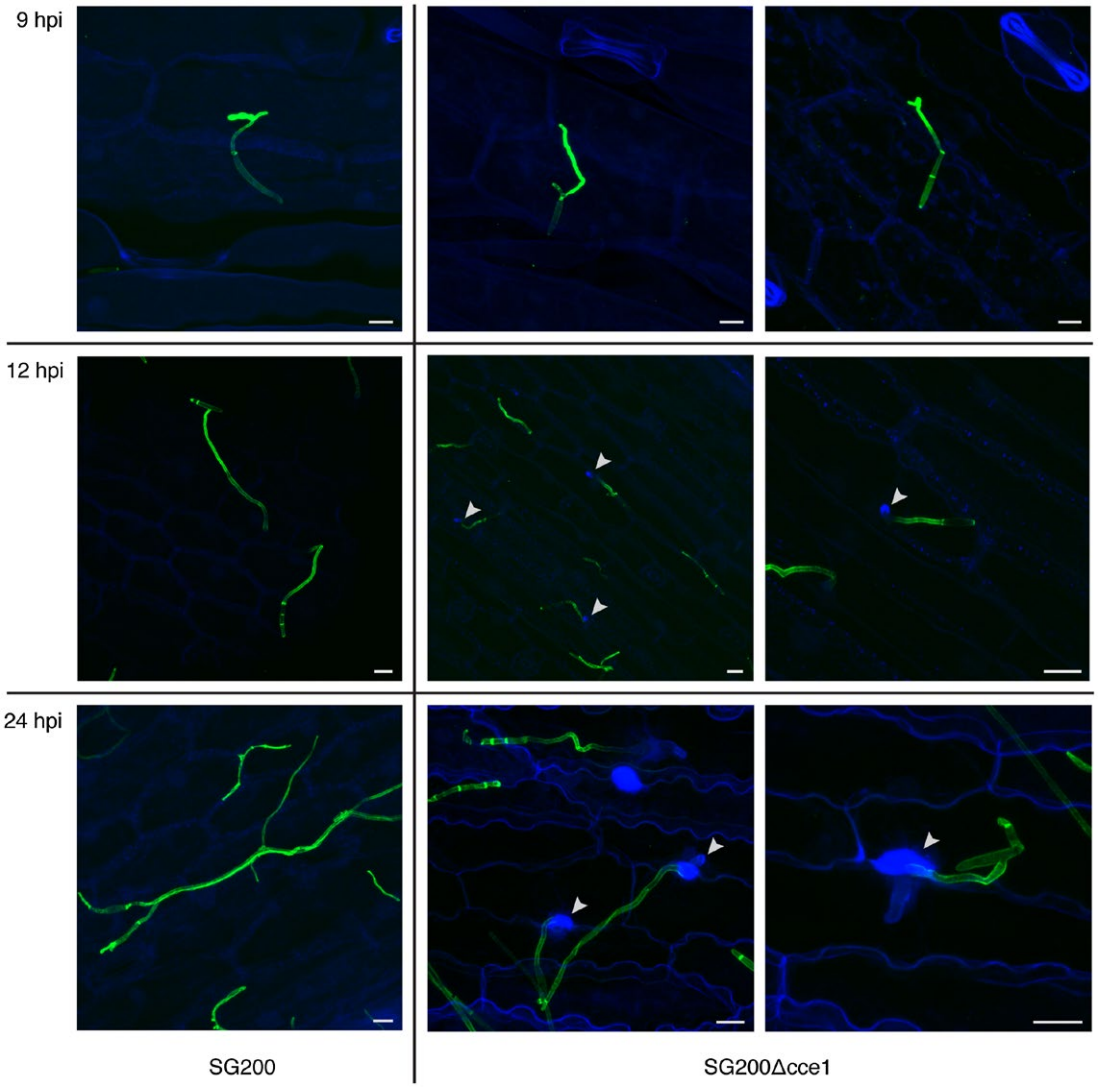
Detection of papillae formation by callose staining with aniline blue. The progenitor strain SG200 (left) does not induce callose deposition, whereas the SG200Δcce1 deletion strain (two right panels) causes papillae formation and callose deposition at penetration sites. Photomicrographs were taken 9, 12, and 24 hours post infection (hpi). WGA‐AF488 fluorescence (green) stains fungal hyphae; aniline blue fluorescence (blue) to visualize callose deposition; white arrows indicate sites of callose deposition. Scale bar = 10 μm.

Plant cell wall modifications, like deposition of papillae enriched with callose ((1,3)‐β‐D‐glucose), are part of the initial defense mechanisms of a plant cell to generate effective barriers at the fungal penetration site (Voigt, 2014). To determine if the plant recognizes the Cce1 deletion strain and if early defense responses are induced, callose staining with aniline blue was performed. No papillae were present in plants infected with the progenitor strain SG200, whereas papillae were visible at the penetration sites of SG200Δcce1 already twelve hours post infection (Fig. 4)*. *At 24 hours post infection, papillae had increased in size and the fungus was hampered in penetrating the plant cell wall. This result indicates that the plant can recognize SG200Δcce1, whereas SG200 is able to suppress the plant’s defense response and inhibit induction of papillae formation.

### Cce1 is conserved among other smut fungi

Homology searches revealed orthologs of *cce1* in the sequenced genomes of the smut fungi *Ustilago bromivora, Ustilago hordei, Sporisorium scitamineum*, and *Sporisorium reilianum *as well as in the ustilaginomycetous anamorphic yeast *Moesziomyces antarcticus* (Laurie et al., 2012; Morita et al., 2014; Rabe et al., 2016a; Schirawski et al., 2010; Taniguti et al., 2015)*. *Alignment of these orthologous proteins showed that the sequences are highly conserved, with sequence identity varying from 59% to 65%. Protein similarity varies between 66% to 74% (Table 1). The part of the secretion signal as well as the C‐terminus are the least conserved regions (Supporting Information Fig. S7). All eight cysteine residues present in the center of the protein are conserved in all of the orthologs, indicating an important role for the putative disulfide bonds. Further, all of the Cce1 orthologs are predicted to be secreted by SignalP and, except for the *S. scitamineum* ortholog, are predicted to localize to the apoplastic space (Table 1) (Petersen et al., 2011; Sperschneider et al., 2017). Interestingly, the neighboring genes of Cce1, UMAG_11489, UMAG_11490, UMAG_02874, and UMAG_11491 are conserved in the genomes of the aforementioned fungi, even in the dicot specific fungus *Melanopsichium pennsylvanicum*, which does not contain an ortholog of Cce1 (Supporting Information Fig. S8 and Table S1).

**Figure 5:**
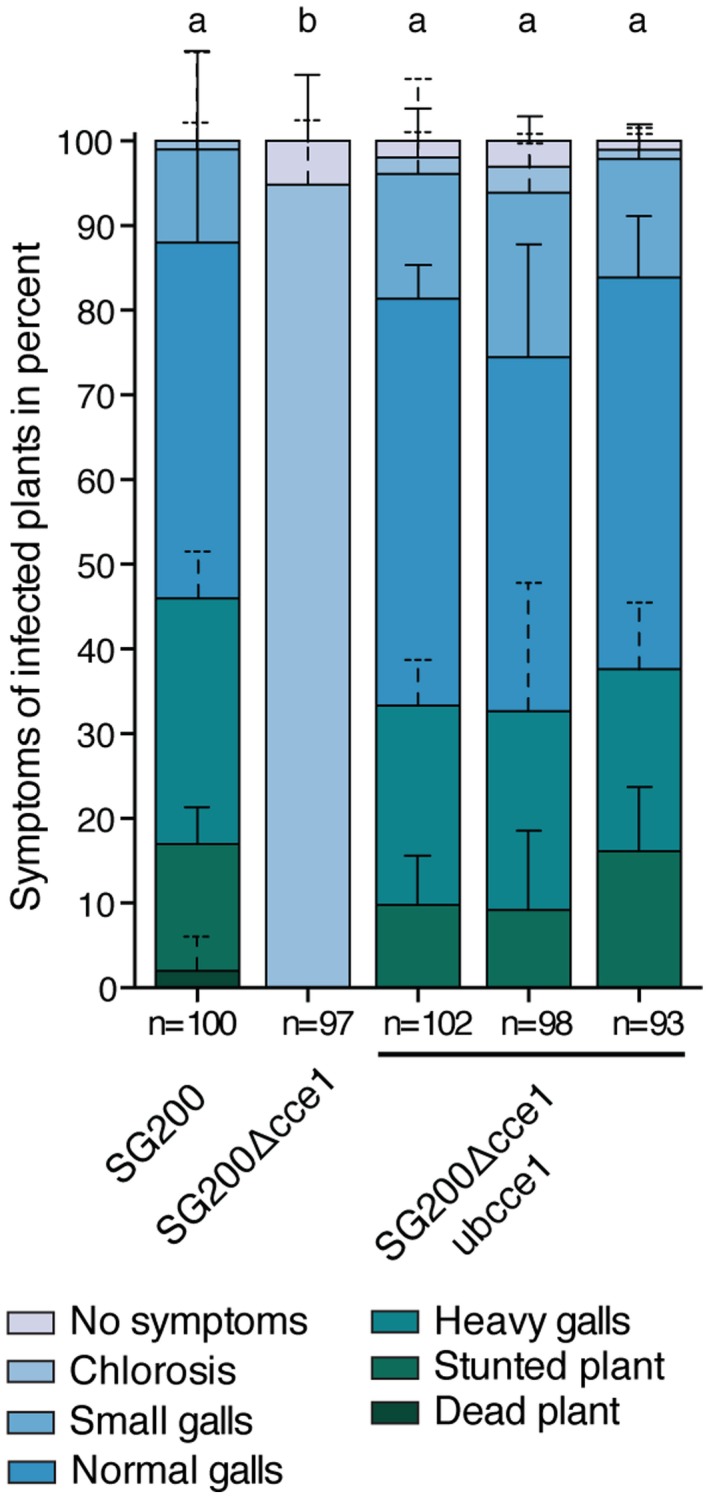
Cce1 complementation with the *U. bromivora* ortholog. Symptom rating of the *U. maydis *strain SG200Δcce1 in comparison to the progenitor strain SG200 and three individual SG200Δcce1‐ubcce1 complementation strains seven days post infection (dpi). Mean and standard deviation of relative counts from three replicates are displayed. For clarity, only positive error bars are shown. n = number of plants scored. Significant differences between the strains are indicated by a, b. p‐values calculated by Fisher’s exact test, MTC by Benjamini‐Hochberg algorithm. α = 0.05.

Given the high similarity between the orthologs of Cce1, we chose the least similar ortholog, Ubcce1 from *U. bromivora* with 66% similarity to *U. maydis*, to perform complementation experiments. We introduced Ubcce1 under control of the *U. maydis cce1 *promoter into the SG200Δcce1 strain*. *Pathogenicity assays revealed that Ubcce1 can fully complement the mutant phenotype of SG200Δcce1 (Fig. 5).

As an essential effector with a high degree of conservation across various smut fungi, Cce1 is a core effector of *U. maydis.*

## Discussion

Over the past several years, major progress has been made in identifying virulence factors and understanding the role of effector proteins (Giraldo and Valent, 2013; Rovenich et al., 2014; Toruno et al., 2016). With the growing number of sequenced genomes of various plant pathogenic microorganisms, the number of putative effectors has grown rapidly. Compared to the number of predicted effectors, there is very little experimental evidence for their functions. In *U. maydis*, only a limited number of effectors show a significant loss of virulence when they are deleted (Doehlemann et al., 2009; Kämper et al., 2006; Mueller et al., 2013; Stirnberg and Djamei, 2016). Among the 467 predicted effector protein coding sequences in the *U. maydis* genome (Schuster et al., 2017), Cce1 has several features of a typical effector. It is relatively small (129 aa) and does not contain any known domains. Moreover, we experimentally confirmed the functionality of its signal peptide and showed that Cce1 is strongly induced during biotrophic stages.

Effector evolution is based on the trade‐off between optimal virulence and avoiding recognition by the host immune receptors. It has been proposed that the high selection pressure on this class of molecules has led to an accumulation of putative effectors in transposon‐rich regions (Rouxel et al., 2011) or other dynamic regions such as chromosomal breakpoints (de Jonge et al., 2013), which enable the accelerated evolution (Schirawski et al., 2010) of these sequences. In smut fungi, effectors are often found in clusters as a result of gene duplication events followed by this accelerated evolution. This has led to a relatively low degree of conservation among smut effectors in general (Kämper et al., 2006; Laurie et al., 2012; Rabe et al., 2016a; Schirawski et al., 2010). In this respect, Cce1 is an atypical smut effector as it is not clustered with other putative effector genes and does not have any paralogs. Its relatively high conservation between smuts that infect diverse monocot hosts and our demonstration that the *U. bromivora* Cce1 ortholog can complement the loss of Cce1 from *U. maydis* indicates that Cce1’s mode of action and target is very likely conserved between *Brachypodium spec.* and maize. Orthologs of *cce1* can be found in the majority of smut fungi, suggesting that Cce1 is a core effector. However, Cce1 is not present in the only sequenced dicot specific smut, *Melanopsichium pennsylvanicum*. This could be because Cce1’s target is monocot specific, leading to its loss in this *Persicaria spec. *infecting smut (Sharma et al., 2014). Alternatively, an evolutionary unrelated but functionally redundant effector evolved in *M. pennsylvanicum *allowing the *cce1* ortholog to be lost from its genome.

The observation that ectopically expressed, tagged versions of Cce1 could only partially complement the virulence defect of the SG200Δcce1 deletion strain could either indicate that these tags partially inhibit the function of the Cce1 protein or that ectopic expression does not exactly mimic the expression of *cce1* from its native locus and that fine tuning of its transcriptional regulation is important for Cce1 function.

Our results, as well as the apoplastic localization prediction by ApoplastP, indicate that Cce1 might act in the biotrophic interphase. Eight highly conserved cysteines in the Cce1 polypeptide are likely to form four disulfide bonds, providing structural stability to the protein under the oxidative environment of the apoplast. The Cce1‐mCherry‐HA fusion protein accumulates in the biotrophic interphase *in vivo* and complements the knockout phenotype. However, as cleavage products of Cce1‐mCherry‐HA were detected by immunoblotting, we cannot exclude that Cce1 might be cleaved and translocated into the plant cell functioning as an symplastic effector. The virulence phenotype of the *cce1* deletion strain shows strong similarities to the phenotype of the deletion strain of pep1, an apoplastic core effector of *U. maydis *(Doehlemann et al., 2009; Hemetsberger et al., 2012, 2015 ). *pep1* deletion strains are apathogenic and are blocked in the first epidermal cell after penetration, as is the Δ*cce1* strain. Both strains induce papillae formation, indicated by callose deposition, as a strong defense response of the host plant (Hemetsberger et al., 2012). In the case of Pep1, host peroxidases involved in PTI have been identified as targets. We have not yet identified targets of Cce1; a yeast two‐hybrid screen as well as immunoprecipitation from maize tissue infected with SG200 cce1‐HA expressing strains followed by mass spectrometry did not identify relevant targets (data not shown). In the yeast two‐hybrid screens, apoplastic localized gene products are underrepresented due to putative mislocalization caused by the signal peptide. Failure of the mass spectrometry approach was possibly due to low amounts of the Cce1‐HA protein. Another explanation is that the target of Cce1 is not a protein, and would therefore not be identified by either yeast two‐hybrid or immunoprecipitation methods.

Given the similarity of the virulence defect phenotype with Δpep1 mutant strains, we speculate that Cce1 inhibits early PTI activation or responses. Identification of the molecular mechanism remains a challenge for future research.

## Experimental procedures

### Strains, plasmids and infection assays

Standard molecular cloning procedures were applied to generate plasmids (Sambrook et al., 1989). Primers and plasmids used in this study are listed in Supporting Information Tables S2 and S3. Plasmids are based on the p123 vector (Aichinger et al., 2003). pUG plasmids were generated using the GreenGate modular system (Lampropoulos et al., 2013) and adapting the p123 vector accordingly. DNA used for the modules of the GreenGate system were either obtained from the published system (Lampropoulos et al., 2013) or directly amplified by PCR (see Supporting Information Table S2 for primers). Vector maps with detailed sequence information are available on request.

*U. maydis *strains were generated by gene replacement via homologous recombination with PCR‐derived constructs (Kämper, 2004) or by ectopic insertion of p123 derivatives into the *ip *locus (Keon et al., 1991; Loubradou et al., 2001). Transformants were confirmed by PCR. *U. maydis *was grown in liquid culture in YEPS light (0.4% yeast extract, 0.4% peptone, 2% sucrose) or on PD plates (2.4% Potato Dextrose Broth, 2% agar) at 28°C. Pathogenicity assays and disease symptom scoring were performed as described (Kämper et al., 2006). To test different stress conditions, serial dilutions of *U. maydis *strains were spotted on CM agar plates containing 1% glucose and different stress‐inducing compounds, and incubated at 28°C for 48 h. For induction of filamentous growth, strains were spotted on CM agar plates containing 1% activated charcoal.

*Zea mays *variety EGB (Old Seeds, Madison, WI, USA) was grown in a temperature‐controlled glasshouse (14 h/10 h light/dark cycle, 28°C/20°C).

### Quantitative real‐time PCR

Quantitative real‐time PCR was performed as described in Rabe *et al*. 2016 (Rabe et al., 2016b). In brief, RNA was extracted from *U. maydis *axenic culture or from infected plant material using the TRIzol method according to the manufacturer’s protocol (Thermo Fisher Scientific, Waltham, MA, USA) followed by DNA removal using DNase I (DNA‐free kit, Thermo Fisher Scientific, Waltham, MA, USA). Reverse transcription was performed using the RevertAid First Strand cDNA Synthesis Kit (Thermo Fisher Scientific, Waltham, MA, USA). The Roche LightCycler® 96 system (Roche Diagnostics, Rotkreuz, Switzerland) was used to perform quantitative real‐time PCR measurements according to manufacturer’s instructions. Relative expression values were calculated using the 2^‐ΔΔCt^ method (Livak and Schmittgen, 2001). Statistical analysis and graphical outputs were performed using GraphPad Prism (Version 6.0, GraphPad Software, La Jolla, CA, USA).

### Secretion assay and immunoblotting

Detection of secreted proteins from fungal hyphae was performed as described in Djamei et al., 2011 (Djamei et al., 2011). In brief, 6 h after filament induction, the cell‐free supernatant was precipitated with 10% trichloroacetic acid (final concentration) and 0.02% sodium deoxycholate (final concentration) and protein extracts of the filamentous cells and culture supernatants were subjected to immunoblotting. For detection of Cce1‐mCherry‐HA fusion proteins infected plant material was grinded and proteins were extracted using the extraction buffer described in Lo Presti et al., 2017 (Lo Presti et al., 2017). Protein extracts were subjected to immunoblotting. Actin detection via mouse anti‐actin antibody (Invitrogen, Waltham, MA, USA) served as a lysis control. Proteins were detected using mouse anti‐haemagglutinin (anti‐HA) antibody (Sigma‐Aldrich, St. Louis, MO, USA).

### Microscopy

For visualization of fungal proliferation in infected tissue, fungal chitin was stained with wheat germ agglutinin (WGA) conjugated to the fluorescent dye Alexa Fluor® 488 (Invitrogen). Plant cell walls were counterstained with propidium iodide (Sigma‐Aldrich, St. Louis, MO, USA). Maize leaves were de‐stained in 100% ethanol. After de‐staining, the leaves were covered with 10% KOH and incubated at 85°C for three to four hours. Samples were washed with 1x PBS until a pH of 7.4 was reached. After washing, the leaves were covered with staining solution (20 µg/ml propidium iodide, 10 μg/ml WGA‐AF488, 0.02% Tween20 in 1x PBS (pH 7.4)) and vacuum was applied three times. Visualization of fluorescently tagged proteins was performed by direct observation by confocal microscopy of infected leaf samples. Appressoria formation and penetration efficiency experiments were performed as described (Mendoza‐Mendoza et al., 2009; Stirnberg and Djamei, 2016). Briefly, two strains (SG200AM1‐RFP and SG200Δcce1‐AM1) were mixed, maize was infected with this cell suspension, and leaf sections were stained 18‐20 hours post infection. SG200AM1‐RFP cells appear red due to expression of RFP (red fluorescent protein) and blue due to calcofluor white staining. After induction of the appressorial marker, SG200AM1 cells appear blue, red, and green due to induction of GFP (green fluorescent protein). SG200Δcce1‐AM1 cells appear blue before appressoria induction or green and blue after. Confocal microscopy was performed using an LSM780 Axio Observer inverted microscope (Zeiss). Aniline blue staining for callose detection was performed by de‐staining infected maize leaves in 100% ethanol. After de‐staining, samples were incubated in 1x PBS for 30 min. Leaves were covered with staining solution (10 μg/ml WGA‐AF488, 0.02% Tween20 in 1x PBS (pH 7.4)) and incubated for 30 min. Samples were washed with 1xPBS and incubated in sodium phosphate buffer (0.07 M, pH 9) for 30 min followed by incubation with 0.005% Aniline blue solution (in sodium phosphate buffer 0.07 M, pH 9) for one hour. Leaves were washed with sodium phosphate buffer and visualized by confocal microscopy. Plasmolysis was induced by infiltrating leaf sections with 1M NaCl (Doehlemann et al., 2009). 3D rendering was performed by applying the 3D Surface Plot function of the ImageJ 2.0.0 software on previously obtained z‐stacks of confocal microscopy images. In this work, the following conditions were used: WGA‐AF488, excitation at 488 nm, detection at 499‐552 nm; propidium iodide, excitation at 561 nm, detection at 595‐665 nm; mCherry, excitation at 561 nm, detection at 578‐648 nm; Aniline blue, excitation at 405 nm, detection at 415‐502 nm; Calcofluor white, excitation at 405 nm, detection at 415‐464 nm; GFP, excitation at 561 nm, detection at 473‐552 nm; RFP, excitation at 488 nm, detection at 578‐657 nm. Microscopy images were processed using the software ZEN 2012 SP1. Filament formation on charcoal plates was observed with a Zeiss widefield stereo microscope.

### Bioinformatic analysis

Gene and protein sequences of *U. maydis, U. hordei, U. bromivora, S. reilianum *and *S. scitamineum *were obtained from the Pedant *Ustilago maydis* Database, the Pedant *Ustilago hordei* Database, the Pedant *Ustilago bromivora *Database, the Pedant *Sporisorium reilianum* Database and the Pedant *Sporisorium scitamineum* Database, respectively (https://pedant.helmholtz-muenchen.de). Sequences of *M. antarcticus* were obtained from the National Center for Biotechnology Information (NCBI). Amino acid sequence alignments were performed using CLC Main Workbench 7 (Qiagen, Hilden, Germany). Protein sequence identity and similarity values were obtained using the Ident and Sim tool from The Sequence Manipulation Suite (https://www.bioinformatics.org/sms2/ident_sim.html) (Stothard, 2000). SignalP 4.1 was used to predict secretion signals (https://www.cbs.dtu.dk/services/SignalP/) (Petersen et al., 2011). For prediction of apoplastic localization, ApoplastP 1.0 was used (https://apoplastp.csiro.au/index.html) (Sperschneider et al., 2017).

An R script was used for disease scoring evaluation as described in Stirnberg *et al*. (Stirnberg and Djamei, 2016). Class counts of biological replicates were summarized and Fisher’s exact test was used to determine significant differences between genotypes. The Benjamini‐Hochberg algorithm was used for multiple testing correction (Benjamini et al., 2001). For visualization, each genotype and replicate was converted to relative counts. GraphPad prism (Version 6.0, GraphPad Software, La Jolla, CA, USA) was used for data visualization.

Statistical analysis and graphical output of appressoria marker was performed using GraphPad prism (Version 6.0, GraphPad Software, La Jolla, CA, USA).

## Conflict of interest

The authors declare that there is no conflict of interest in the research.

## Supporting information

Additional supporting information may be found in the online version of this article at the publisher's web‐site
