# Supplementary figures and images for: The core effector Cce1 is required for early infection of maize by Ustilago maydis
Source: Mol Plant Pathol. 2018 Aug 16;19(10):2277–87. doi: 10.1111/mpp.12698 (PMC6638113; doi:10.1111/mpp.12698)

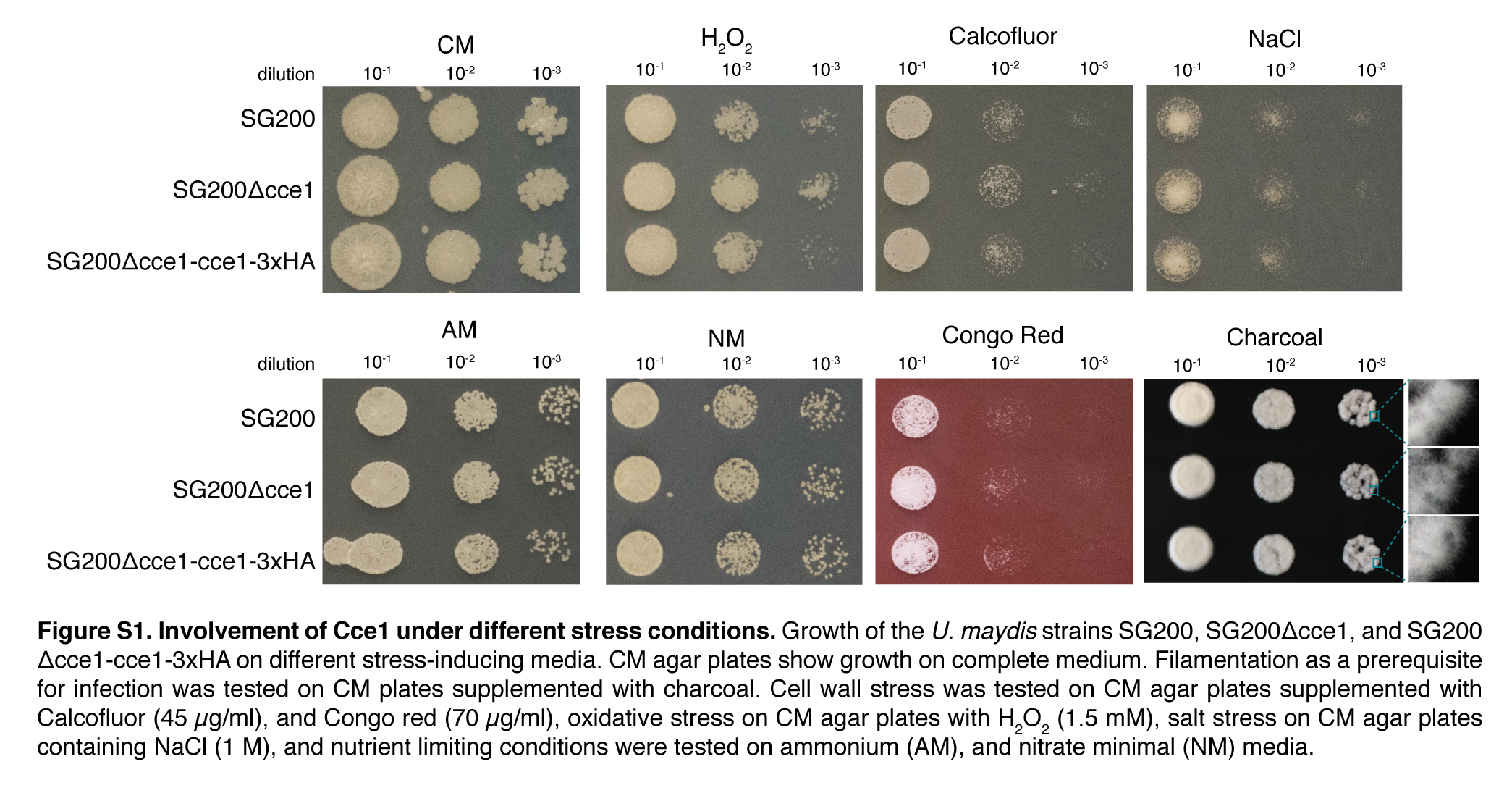

Supplement: Supplementary file 1 [file MPP-19-2277-s001.tif]

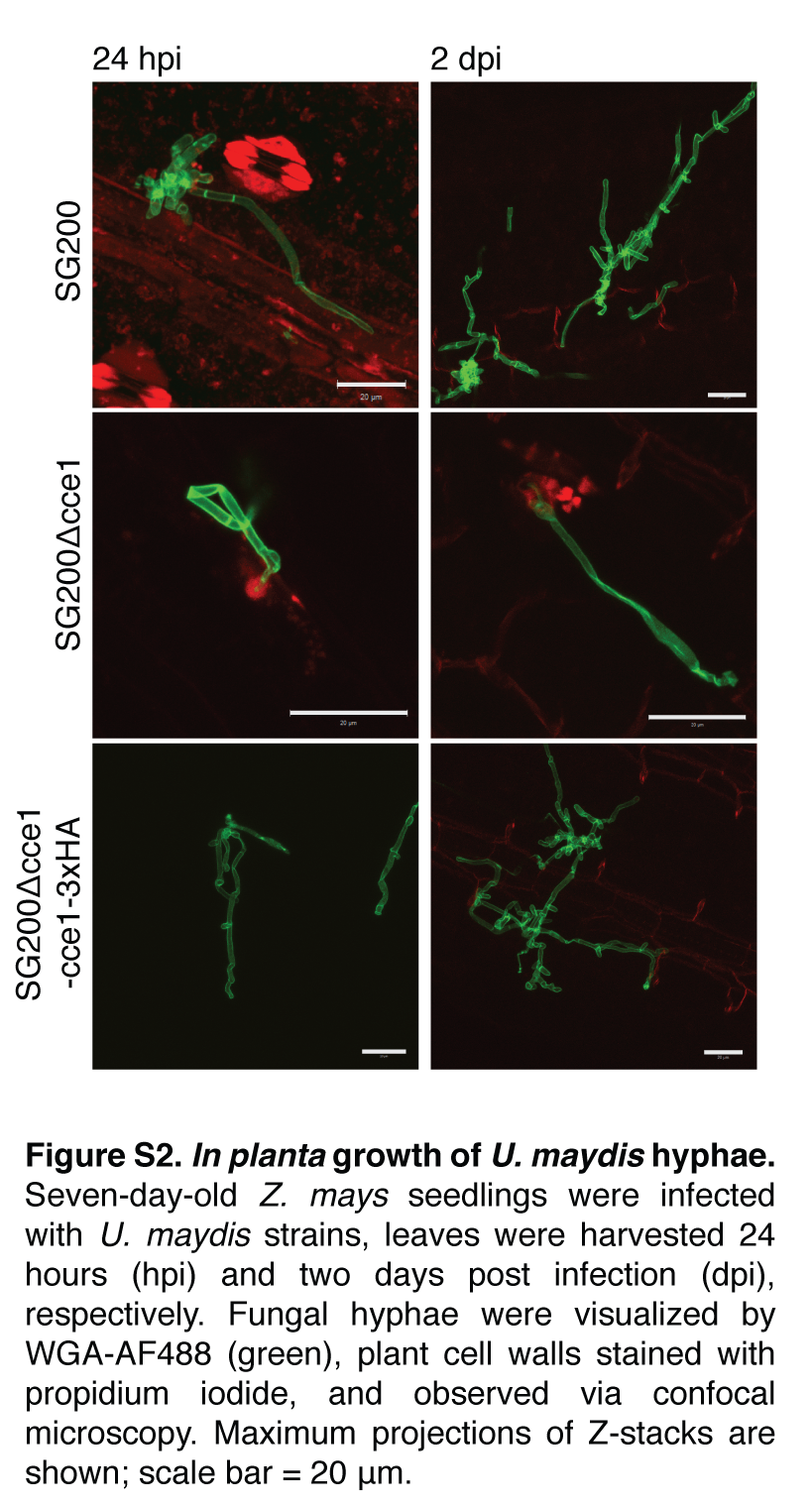

Supplement: Supplementary file 2 [file MPP-19-2277-s002.tif]

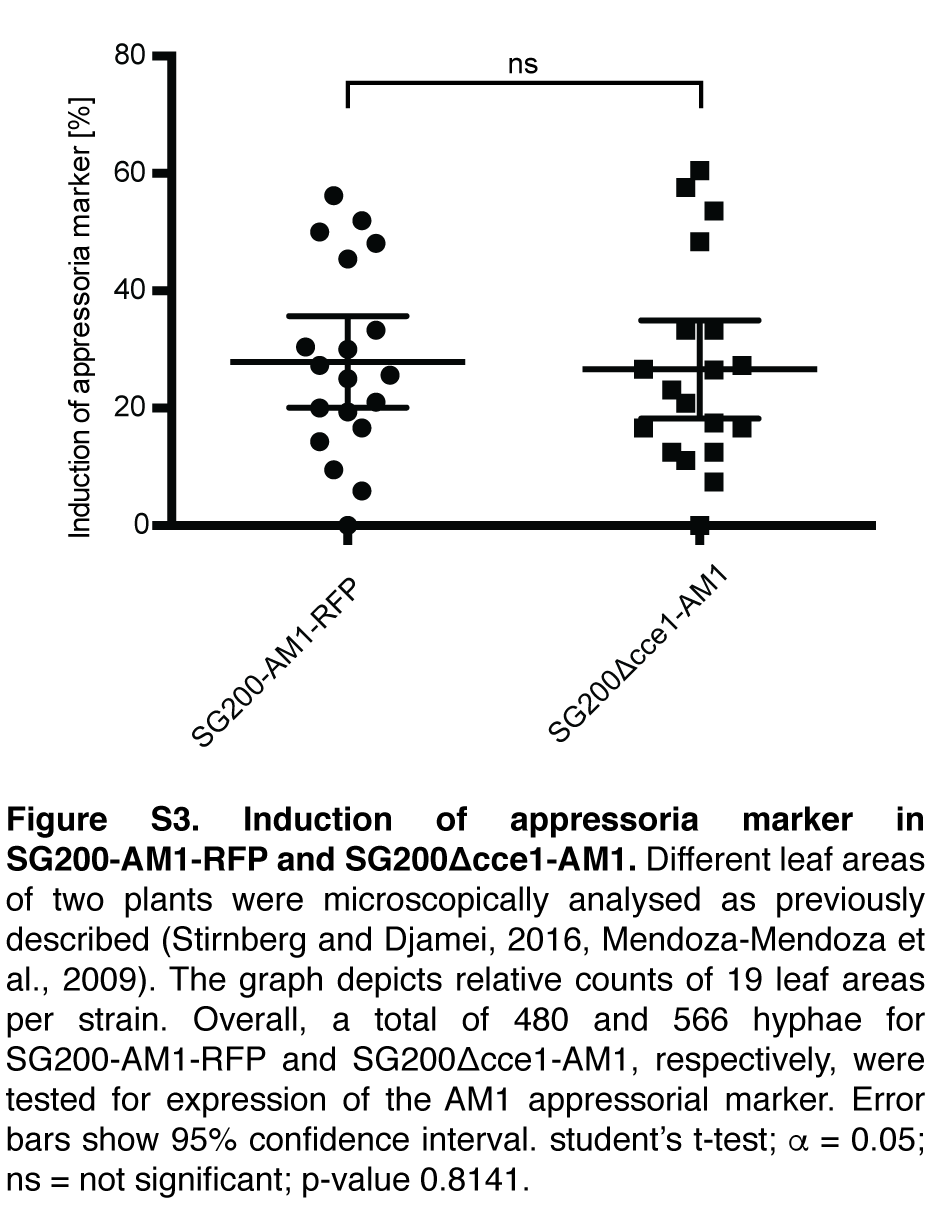

Supplement: Supplementary file 3 [file MPP-19-2277-s003.tif]

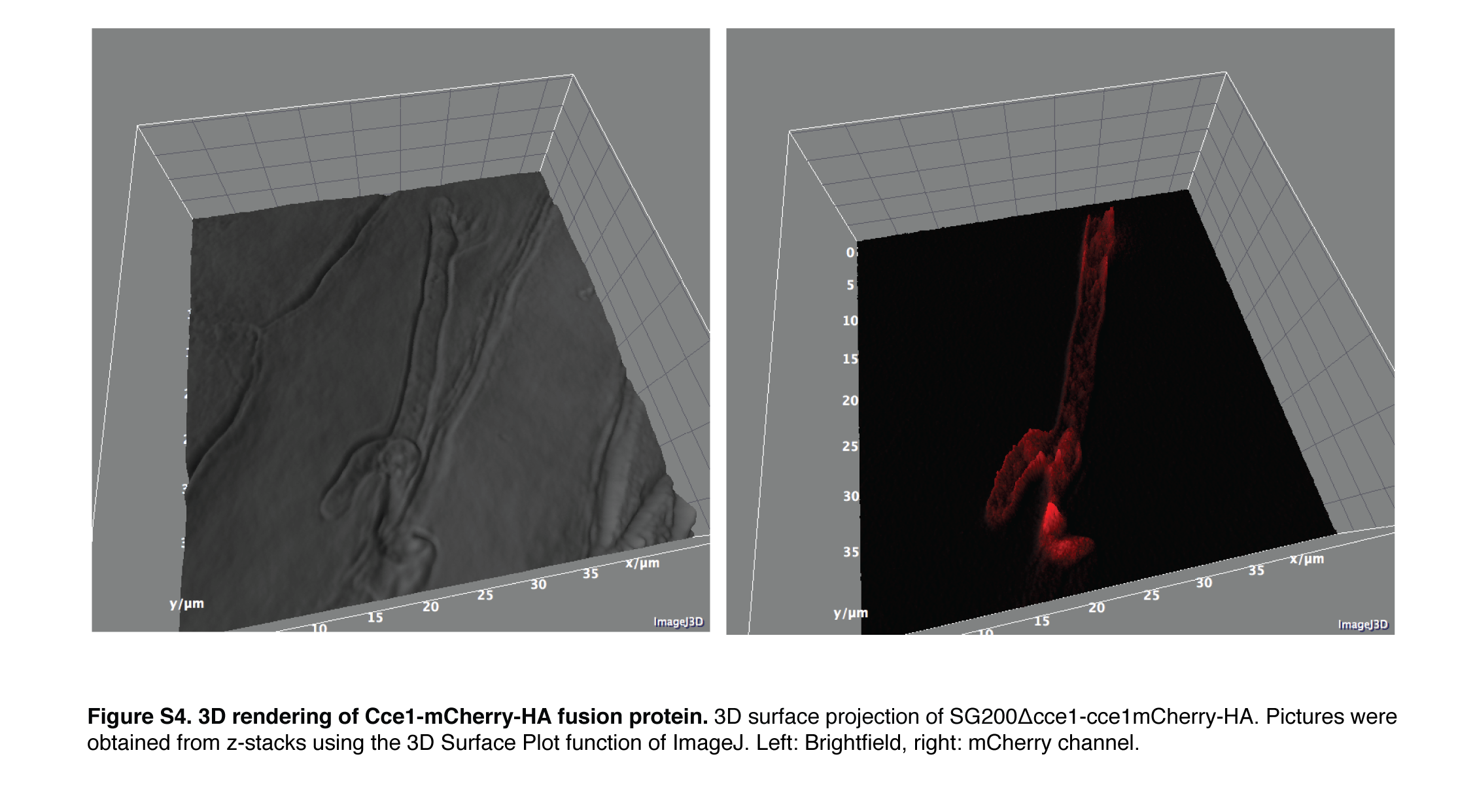

Supplement: Supplementary file 4 [file MPP-19-2277-s004.tif]

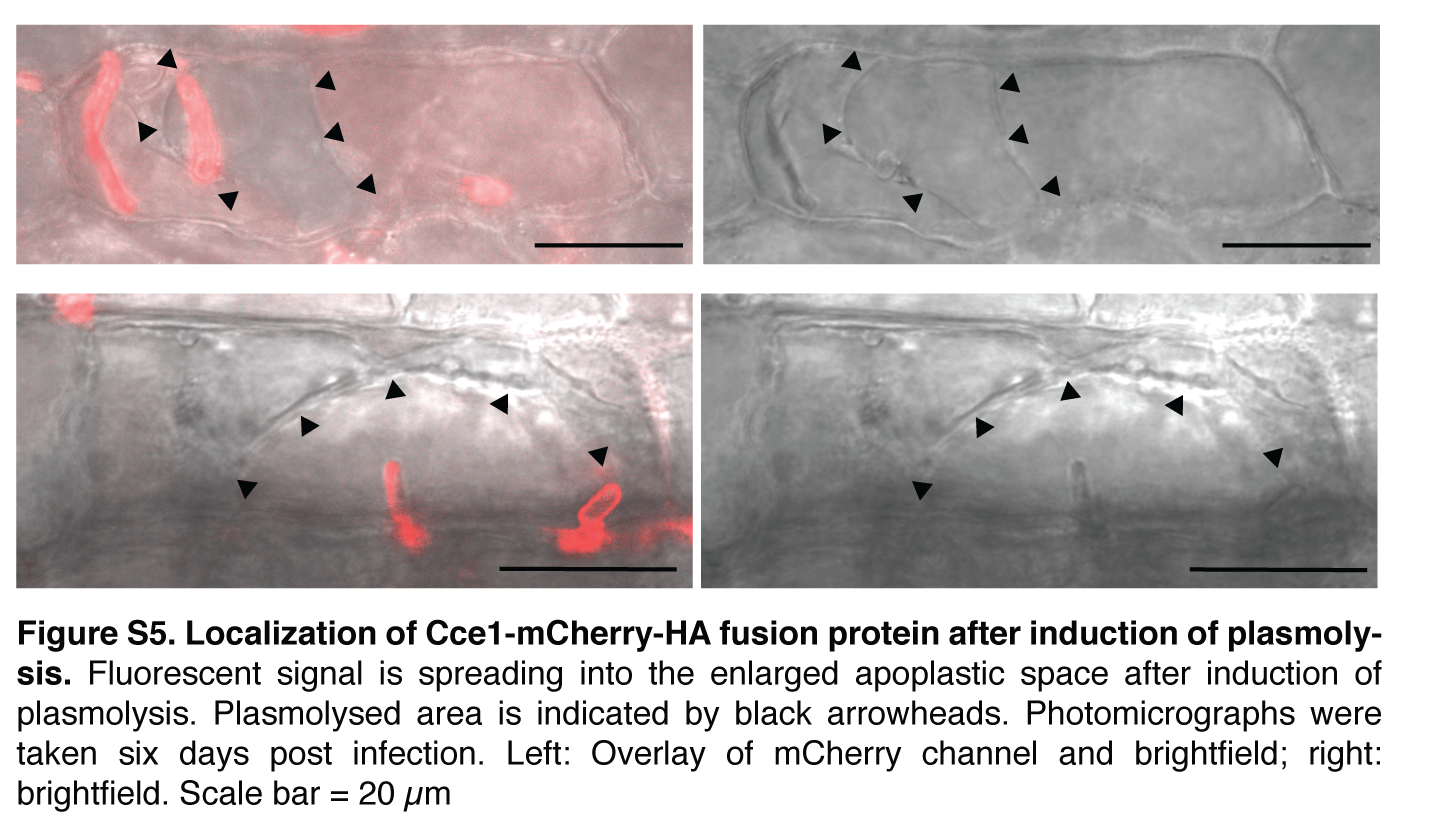

Supplement: Supplementary file 5 [file MPP-19-2277-s005.tif]

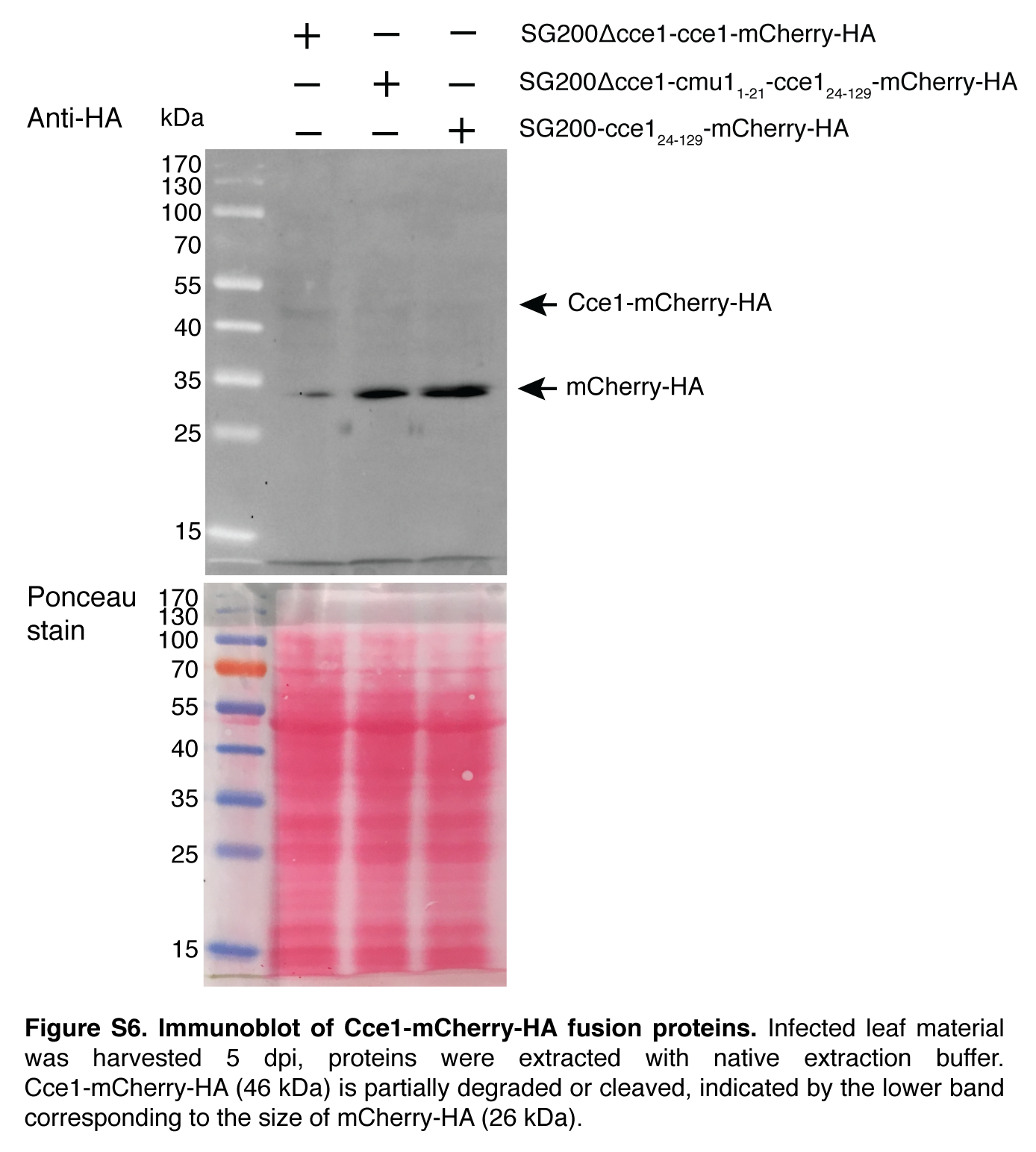

Supplement: Supplementary file 6 [file MPP-19-2277-s006.tif]

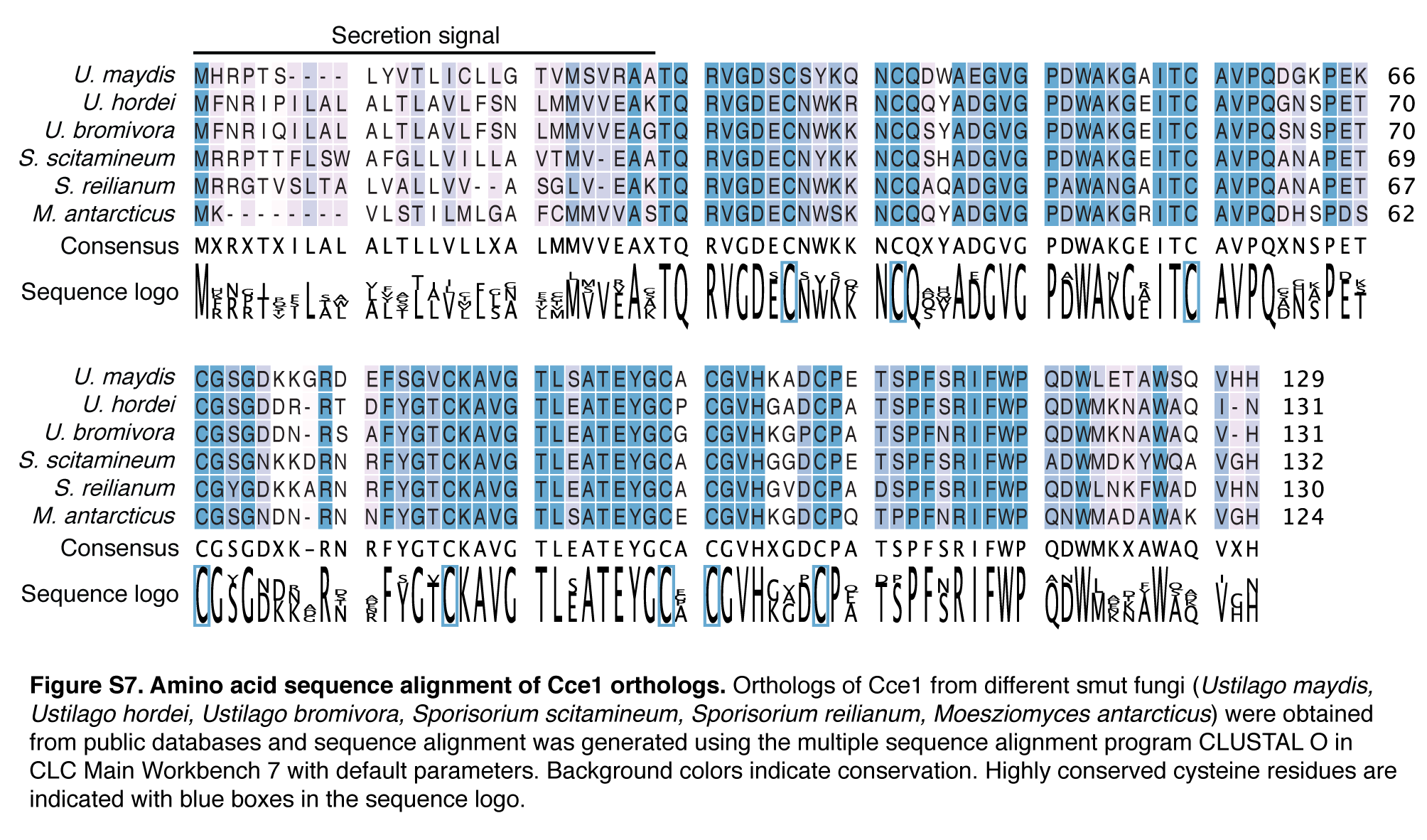

Supplement: Supplementary file 7 [file MPP-19-2277-s007.tif]

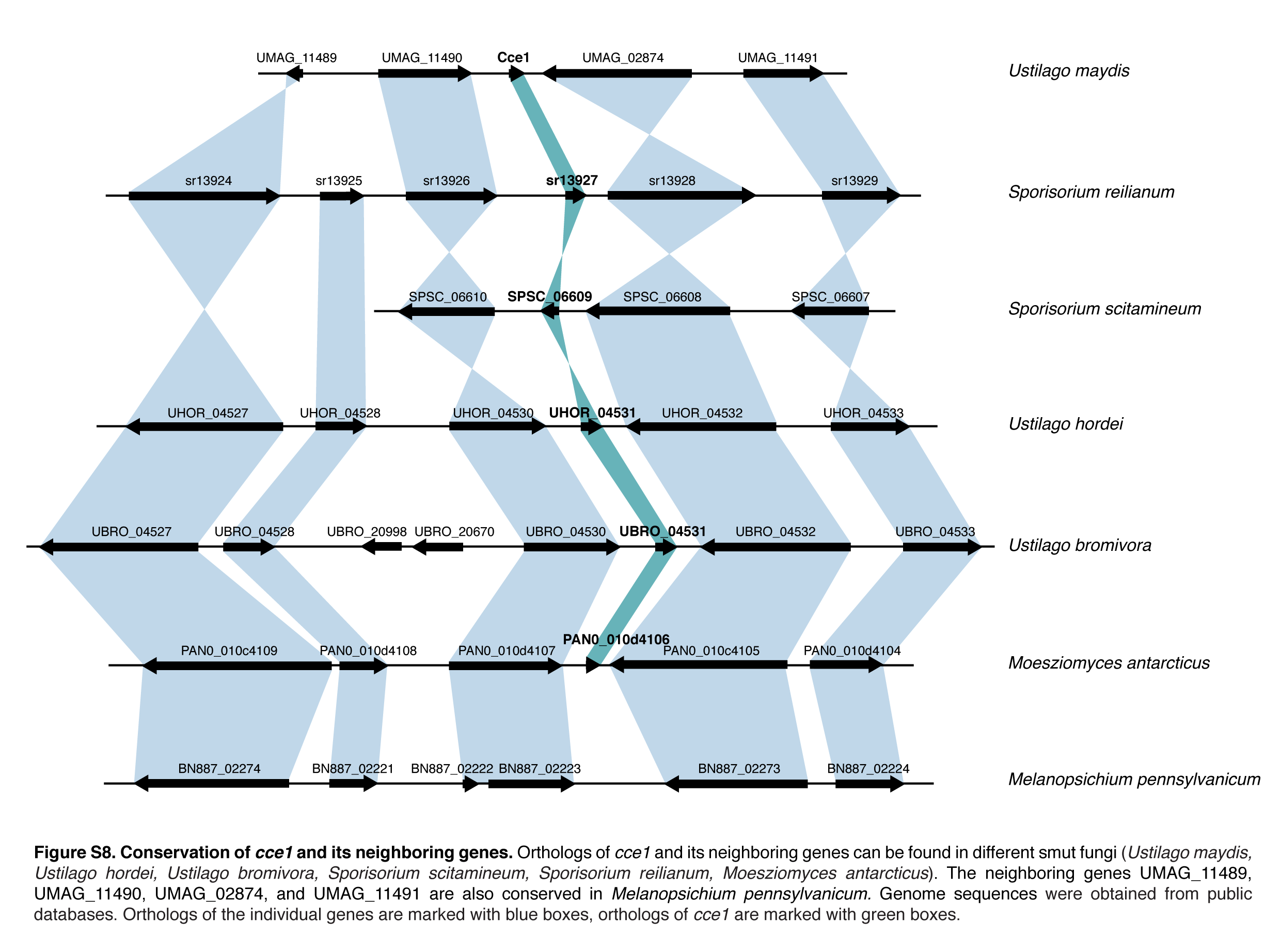

Supplement: Supplementary file 8 [file MPP-19-2277-s008.tif]
